# Lysosomal lipid peroxidation mediates immunogenic cell death

**DOI:** 10.1172/JCI169240

**Published:** 2023-04-17

**Authors:** Pravin Phadatare, Jayanta Debnath

**Affiliations:** Department of Pathology and Helen Diller Family Comprehensive Cancer Center, University of California, San Francisco, San Francisco, California, USA.

## Abstract

Cancer cells rely on lysosome-dependent degradation to recycle nutrients that serve their energetic and biosynthetic needs. Despite great interest in repurposing the antimalarial hydroxychloroquine as a lysosomal inhibitor in clinical oncology trials, the mechanisms by which hydroxychloroquine and other lysosomal inhibitors induce tumor-cell cytotoxicity remain unclear. In this issue of the *JCI*, Bhardwaj et al. demonstrate that DC661, a dimeric form of chloroquine that inhibits palmitoyl-protein thioesterase 1 (PPT1), promoted lysosomal lipid peroxidation, resulting in lysosomal membrane permeabilization and tumor cell death. Remarkably, this lysosomal cell death pathway elicited cell-intrinsic immunogenicity and promoted T lymphocyte–mediated tumor cell clearance. The findings provide the mechanistic foundation for the potential combined use of immunotherapy and lysosomal inhibition in clinical trials.

## Lysosomal inhibition as a therapeutic approach for cancer

**Figure 1:**
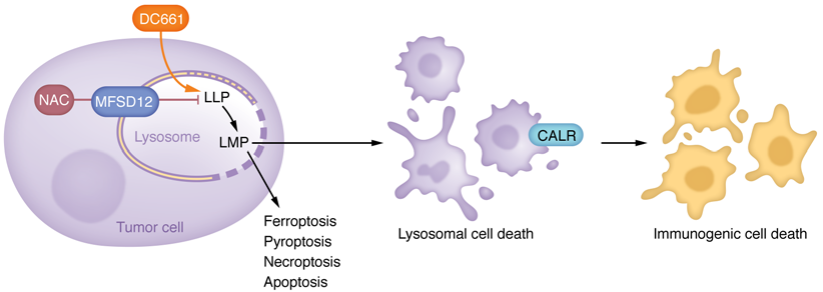
DC661 induces lysosomal lipid peroxidation and immunogenic cell death. The PPT1 inhibitor DC661 promotes LLP resulting in LMP and immunogenic cell death, marked by cell surface expression of CALR. DC662-mediated LLP can be reversed by NAC, which is transported into lysosomes via the cysteine transporter MFSD12.

Lysosomes are membrane-bound vesicles containing hydrolytic enzymes that degrade diverse cellular contents, including proteins, carbohydrates, and lipids (1). In tumors, there is a growing recognition that lysosome-dependent degradation is a crucial mediator of nutrient recycling necessary to sustain both the energetic and biosynthetic demands of the cancer cell (2, 3). Notably, two lysosomal-dependent nutrient scavenging pathways, autophagy and macropinocytosis, critically promote the survival and metabolic adaptation of tumor cells (1, 2). As a result, lysosomal inhibition has been proposed as a promising therapeutic approach to treat diverse cancers. Most notably, this approach is exemplified through the repurposing of the antimalarial hydroxychloroquine (HCQ) in diverse clinical oncology trials (2). However, the results of these HCQ trials have been mixed, which has prompted the need for next-generation lysosomal inhibitors and a more thorough understanding of the mechanisms directing the anticancer properties of these lysosomal inhibitors. Although HCQ and other chloroquine derivatives were originally proposed to deacidify the lysosome and promote lysosome membrane permeabilization (LMP), multiple studies by the Amaravadi lab identified palmitoyl-protein thioesterase 1 (PPT1) as the molecular target of chloroquine and its derivatives (4, 5). Building on this elegant work, Bhardwaj et al. report in this issue of *JCI* that PPT1 inhibition promotes lysosomal lipid peroxidation (LLP), resulting in lysosomal membrane permeabilization and tumor cell death (Figure 1) (6).

## Lysosome inhibition induces immunogenic cell death

Bhardwaj and colleagues primarily analyzed the response of tumors cells to DC661, a dimeric form of chloroquine and next-generation lysosomal inhibitor that is more efficient than HCQ in its ability to penetrate cancer cells and localize to the lysosome (5). DC661 potently blocked autophagy and induced multiple cell death pathways, including apoptosis, necroptosis, ferroptosis, and pyroptosis (Figure 1). However, none of these major death pathways were individually required for cytotoxicity. Furthermore, pharmacological inhibitors of cathepsin- and calcium-dependent lysosomal death pathways also did not prevent cell death. Rather, DC661 induced LLP resulting in LMP, which led to lysosomal cell death in tumor cells. Consistent with a role for ROS as the inducing mechanism for lipid damage, DC661 induced lysosomal lipid peroxidation that could be reversed with the ROS scavenger N-acetylcysteine (NAC), which potently attenuated both lysosomal membrane permeabilization and cytotoxicity in multiple cancer cell lines. Importantly, NAC also reversed LMP that was induced by genetic knockdown of PPT1 or treatment with high concentrations of HCQ. Notably, NAC was the only antioxidant able to reverse DC661-induced cell death, which completely depended on the presence of the lysosomal cysteine transporter MFSD12 (Figure 1). Other commonly employed antioxidants, such as Trolox and vitamin C, were unable to prevent LLP or the cytotoxic effects induced by DC661, possibly due to their lack of adequate lysosomal penetration (6).

Bhardwaj and colleagues also uncovered an unexpected role for LLP in the control of tumor immunity (6). Because HCQ cotreatment sensitizes tumors to immune checkpoint blockade therapy in preclinical models of pancreatic ductal adenocarcinoma, there is great interest in lysosomal inhibition as a strategy to enhance tumor immunity (7). Importantly, recent studies implicate the autophagy pathway in the selective degradation of both MHC class I and the immunoproteasome; accordingly, in certain tumor cell types, autophagy inhibition has been demonstrated to restore surface MHC class I levels and antigen processing (7, 8). However, the administration of DC661 to B16F10 melanoma or MC38 colon adenocarcinoma models did not result in increased surface expression of MHC class I or upregulate the immunoproteasome (6), hence illuminating the cell-type specificity for these previously described immunomodulatory effects of inhibiting autophagy or the lysosome. Instead, DC661-induced LLP and LMP elicited increased cell surface expression of the immunogenic cell death marker calreticulin (CALR), which resulted in enhanced T cell–mediated cytotoxicity in vitro (9). Importantly, either cotreatment with NAC or RNAi-mediated depletion of CALR was sufficient to attenuate DC661-primed T cell cytotoxicity, corroborating the importance of LLP in mediating immunogenic cell death. Furthermore, in vivo vaccination studies using two distinct syngeneic colon adenocarcinoma tumor models demonstrated that DC661-treated cells could promote the rejection of implanted tumors. However, consistent with previous studies using genetic autophagy inhibition, these vaccine-like effects were not observed with DC661-treated B16F10 melanoma cells, despite the upregulation of CALR surface expression (10). Based on these results, Bhardwaj and colleagues postulated that although LLP-mediated lysosomal cell death could induce tumor cell–intrinsic immunogenicity, these changes by themselves may be insufficient to convert a so-called “immune cold” tumor microenvironment into an “immune hot” tumor microenvironment.

## Conclusions and implications

The results from Bhardwaj et al. deepen our understanding of how chloroquine derivatives mediate tumor cell cytotoxicity and illuminate the importance of lysosomal lipid peroxidation as a mediator of immunogenic cell death. The studies also broach questions regarding this lysosomal cell death pathway. First, how does PPT1 inhibition produce lysosomal ROS and lipid peroxidation? Second, because DC661 treatment results in accumulation of autophagy cargo receptors, such as SQSTM1/p62 and TAX1BP1, do secretory autophagy pathways counteract the proteostatic defects observed in response to PPT1 inhibition (6, 11)? Recent studies have demonstrated that lysosomal inhibition activates secretory autophagy to release accumulated autophagic cargo receptors extracellularly, which may explain the variability in intracellular protein increases observed across cancer cell lines in the Bhardwaj et al. study (6, 12). Accordingly, if autophagy cargo receptors are secreted during DC661-mediated lysosomal inhibition, one can speculate that these proteins may serve as noninvasive biomarkers to monitor the efficacy of PPT1 inhibition during cancer treatment. Finally, from the standpoint of therapeutic development, the findings in Bhardwaj et al. broach the intriguing hypothesis that LLP-mediated lysosomal cell death will be most effective in combination with immunomodulatory therapies that enhance T cell infiltration into the tumor microenvironment (6).

## Version 1 04/17/2023

Electronic publication
